# Livedoid Dermatitis Treated With Nifedipine

**DOI:** 10.1177/2324709616629786

**Published:** 2016-02-02

**Authors:** Lee Wheless, Lilly Zhu, Mona Mashayekhi, Rachel B. Fissell

**Affiliations:** 1 Vanderbilt University, Nashville, TN, USA

**Keywords:** livedoid dermatitis, substance-related disorders

## Abstract

Intravenous injection of buprenorphine as a cause of livedoid dermatitis is a recently described phenomenon. This report reviews the brief literature of this finding, and presents a case of livedoid dermatitis of both heels following injection more than one day prior, and thesuccessful treatment with nifedipine monotherapy.

## Introduction

Intravenous injection of buprenorphine as a cause of livedoid dermatitis is a recently described phenomenon.^1,2^ This report presents a successful treatment of a case of buprenorphine-induced livedoid dermatitis with nifedipine monotherapy.

## Case Presentation

**Figure 1.**
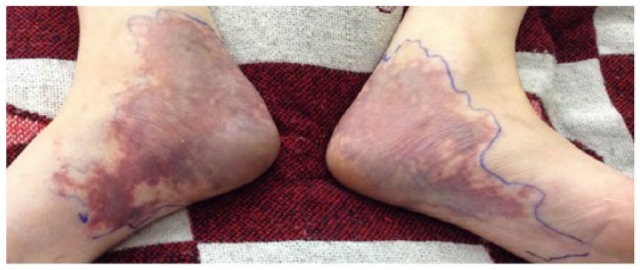
Hospital day 2 with development of classic livedoid appearance, increased edema.

A 34-year-old Caucasian male with past medical history notable for intravenous drug abuse was admitted to the general medicine service with chief complaint of rash, pain, and edema in his heels for the past 1 to 2 days concerning for cellulitis. The patient reported he had been unable to find veins in his upper extremities, and so had been injecting buprenorphine into his posterior tibial veins. The rash had started on his left heel and shortly thereafter appeared on his right heel in a symmetric distribution. The exam on admission was notable for a slightly purpuric mottled erythema that blanched with residual petechiae on his heels and plantar arches. The patient had a history 1 month prior of gangrenous infection of the penis that grew methicillin-resistant *Staphylococcus aureus* following similar injection into the dorsal vein of his penis. He was therefore started on empiric clindamycin. The rash continued to evolve with increased pain and swelling, with the development of a reticular, nonblanching border (Figure 1). The patient had remained afebrile, with no leukocytosis, no increased warmth of the eruption, and negative blood cultures. At this time, it was felt this was not a cellulitis, but rather livedoid dermatitis secondary to injection of buprenorphine due to the clinical appearance and history.^1,2^ Labs drawn during the patient’s previous admission 1 month prior were negative for coagulopathy or vasculitis, including HIV, hepatitis B and C serologies, as were repeat viral serologies. A full vasculitis workup was not repeated during this admission. On hospital day 3 there was further increased erythema, edema, and pain with concern for impending ulceration.^3^ One hypothesis for the underlying mechanism of livedoid dermatitis is local vasospasm.^4,5^ Because of its utility in treating Raynaud’s phenomenon, which is also thought to be caused by local vasospasm, a trial of nifedipine was started. The patient’s clinical presentation slowly improved over the following 2 days and was discharged home with follow-up appointments made with psychiatry and his primary care physician. Despite these efforts, he has been lost to follow-up.

## Discussion

Livedoid dermatitis is an uncommon and preventable complication resulting from inadvertent intra- or peri-arterial injections leading to inflammation, vasospasm, or embolism of insoluble particulates from injections.^4,5^ Recently, this has been recognized among intravenous drug abusers, with case series published in France showing that livedoid dermatitis occurs more often in individuals with prior complications from intravenous drug use.^6,7^ Biopsies of the skin have suggested that, in some cases, emboli of the starch from the crushed buprenorphine tablets occludes the microvasculature in the affected area, leading to local inflammation and necrosis.^6^ As such, treatment modalities have tended to focus on reperfusion and vasodilatation, usually requiring intravenous medications, and often surgical intervention.

Multiple case reports have described treatment with a combination of vasodilatory drugs such as pentoxiphylline or alprostadil, heparin, or systemic steroids.^4,8^ If the disease progresses to necrosis with ulceration, early debridement has been recommended to prevent secondary infection, a complication already seen in this patient on a prior admission.

Skin changes typically present within hours to days, with a mean time to skin necrosis of 12 days.^7^ Our patient’s story fits well within the expected timeframe, with likely necrosis of skin. Given the concern for impending ulceration, a confirmatory biopsy was not obtained. Roughly one third of the patients in a small case series had ulceration requiring surgical repair, while others resolved with scarring, and still others with no lasting effects. While the natural course of the lesion is unknown, the patient’s lesions did show clinical improvement coinciding with treatment with nifedipine, and a clinical effect cannot be excluded.

Several aspects of this case warrant further consideration. First, this form of livedoid dermatitis presents in a subacute timeframe, as opposed to the near-immediate pain and necrosis seen in classic Nicolau syndrome. The classical presentation also occurs more often following intramuscular injections, with several medications implicated, including nonsteroidal anti-inflammatory drugs, certain antibiotics, and notably a case was reported following a patient’s self-injection of the tumor necrosis factor-α inhibitor etanercept.^9,10^ The delayed onset thus can present as a diagnostic dilemma, often being mistaken for a cellulitis in its initial stages, as it was in this case. Second, the later presentation also makes the question of vasodilation and reperfusion less straightforward. In instances where hyperacute presentation occurs, the immediate obstruction and ischemia necessitate an immediate response to restore perfusion to the affected area. However, in patients who present days after the inciting injection, irreversible damage should have already occurred if a similar occlusive event had happened. Skin biopsies in other cases have shown microemboli of the starch particles, indicating that vascular obstruction plays a role; however, to what degree is still unknown. Finally, the bilateral presentation in this case is uncommon. However, with the known history of injection into both posterior tibial veins within a 12- to 24-hour period, it seems likely that the same preparation of drug was injected at both sites with the same result.

## Conclusions

Livedoid dermatitis following buprenorphine injection is a recently described phenomenon with a delayed presentation compared to other causes of this disease. While the exact mechanism is not known, evidence suggests (*a*) emboli of particulate matter from the crushed pills, (*b*) local vasospasm, and (*c*) local inflammation. Treatment of cases usually involves reperfusion with anticoagulation and vasodilators. Lesions that ulcerate often require surgical repair. Herein we describe a case of buprenorphine-induced livedoid dermatitis that was treated with nifedipine monotherapy whose clinical course showed improvement coinciding with nifedipine imitation.
